# Indirect Pulp Treatment for Deep Carious Lesions in Mature Permanent Teeth: A Preventive, Minimally Invasive Clinical Approach

**DOI:** 10.3390/dj14040200

**Published:** 2026-04-01

**Authors:** Liana Beresescu, Alexandra Mihaela Stoica, Csinszka Andrea Kovacs-Ivacson, Timea Dakó, Alexandru Vlasa, Csilla Benedek, Gabriela Felicia Beresescu, Andrea Bors

**Affiliations:** Faculty of Dental Medicine, University of Medicine and Pharmacy, Science, and Technology George Emil Palade, 540139 Târgu-Mureș, Romania; liana.beresescu@umfst.ro (L.B.); andrea.kovacsivacson@umfst.ro (C.A.K.-I.); timea.dako@umfst.ro (T.D.); alexandru.vlasa@umfst.ro (A.V.); csilla.benedek@umfst.ro (C.B.); felicia.beresescu@umfst.ro (G.F.B.); andrea.bors@umfst.ro (A.B.)

**Keywords:** indirect pulp treatment, Biodentine, resin-modified glass ionomer cement, bioactive materials, pulp vitality

## Abstract

**Background:** Indirect pulp treatment (IPT) is a conservative approach aimed at preserving pulp vitality in teeth with deep carious lesions. In adult patients, however, evidence comparing different liner materials remains limited. **Objectives:** To assess the 12-month clinical and radiographic outcomes of IPT performed with Biodentine and resin-modified glass ionomer cement (RMGIC) in mature permanent teeth. **Methods:** A split-mouth clinical study was conducted in adult patients presenting with deep carious lesions in vital permanent teeth. Following selective caries removal, IPT was completed using Biodentine or RMGIC under a standardized operative protocol. Clinical and radiographic evaluations were performed at baseline and after 12 months. Outcomes included pulp vitality, postoperative pain, and radiographic changes. **Results:** At 12 months, pulp vitality was preserved in over 90% of treated teeth in both groups, with no statistically significant differences between materials. Teeth treated with Biodentine showed lower postoperative pain scores at 24 h compared with those treated with RMGIC, although this difference resolved within the first postoperative week. Radiographic outcomes were comparable between groups, with a low incidence of periapical changes. **Conclusions:** Within the limits of this interim analysis, IPT performed with either Biodentine or RMGIC resulted in similar clinical and radiographic outcomes in mature permanent teeth. These findings indicate that pulp vitality preservation can be achieved using different liner materials when minimally invasive principles and effective coronal sealing are applied. Extended follow-up is required to evaluate long-term durability.

## 1. Introduction

Dental caries remains one of the most prevalent chronic diseases worldwide, despite major advances in preventive strategies. According to the World Health Organization, more than 2.4 billion people are affected by untreated carious lesions in permanent teeth, highlighting the persistent global burden of this condition [1]. From a clinical perspective, deep carious lesions represent a particularly challenging scenario, as the clinician must balance caries removal with the risk of irreversible pulpal damage and the subsequent need for invasive treatments such as endodontic therapy or tooth extraction [2].

Beyond their biological implications, deep carious lesions also generate substantial economic and social consequences. Untreated dental pain is associated with missed school days and impaired academic performance in children and adolescents [3], as well as work absenteeism and reduced productivity in adults [4]. In the absence of conservative intervention, deep carious lesions may progress toward irreversible pulpal involvement, ultimately requiring more complex and costly treatments [5]. At the population level, oral diseases account for more than 300 billion USD annually in direct costs, with additional losses due to reduced productivity and absenteeism [6]. These data reinforce the importance of conservative therapeutic approaches capable of preserving tooth vitality while minimizing long-term costs.

Within this context, indirect pulp treatment (IPT) has gained renewed attention as a minimally invasive strategy for managing deep carious lesions. Rather than complete caries excavation, IPT relies on selective removal of infected dentin while preserving a layer of affected but firm dentin adjacent to the pulp, followed by the placement of a protective liner and a well-sealed coronal restoration [2,7]. This approach aims to maintain pulp vitality, reduce the risk of accidental pulp exposure, and support biological healing processes such as tertiary dentin formation. The clinical success of IPT depends not only on the operative technique but also on the liner material used beneath the definitive restoration.

Traditional materials such as calcium hydroxide have demonstrated antibacterial effects and the ability to induce reparative dentin; however, their high solubility and lack of adhesion may compromise long-term outcomes [5]. Glass ionomer cements improved dentin adhesion and fluoride release, although their limited mechanical strength restricted broader indications. Resin-modified glass ionomer cements (RMGICs) were subsequently introduced to improve handling and mechanical properties and remain widely used in everyday clinical practice as pragmatic liner materials [3].

More recently, calcium silicate–based materials such as Biodentine have been proposed as bioactive alternatives. Biodentine exhibits favorable biological properties, including ion release, formation of a mineralized interface with dentin, and stimulation of odontoblastic activity [8]. Clinical studies and systematic reviews have reported high success rates for Biodentine in vital pulp therapy, particularly in younger patients [9,10,11,12]. Most available evidence, however, is derived from single-material evaluations or pediatric and adolescent populations, while data on mature permanent teeth—where pulpal reparative capacity is reduced—remain limited [7,9,10,11,12].

In routine adult dental practice, clinicians frequently choose between bioactive materials such as Biodentine and pragmatic, well-established liners such as RMGIC. Despite their widespread use, direct clinical comparisons between these materials in indirect pulp treatment of mature permanent teeth are scarce, particularly when standardized operative protocols and mid-term follow-up are considered.

Within contemporary caries management concepts, preserving pulp vitality through minimally invasive restorative strategies is increasingly recognized as a key preventive objective.

Therefore, the aim of the present study was to evaluate the clinical performance of two liner materials—Biodentine and resin-modified glass ionomer cement—in indirect pulp treatment of deep carious lesions in mature permanent teeth over a 12-month follow-up period.

### 1.1. Objectives

#### 1.1.1. Primary Objective

To compare the proportion of teeth maintaining clinical and radiographic treatment success at 12 months after indirect pulp treatment using Biodentine versus resin-modified glass ionomer cement (RMGIC).

#### 1.1.2. Secondary Objectives

- To assess postoperative pain intensity during the early postoperative period (24 h and 7 days) and the need for analgesics.
- To compare the integrity and performance of coronal restorations between two liner materials.
- To document tooth discoloration and other restorative complications (recurrent sensitivity or restoration failure).

The null hypothesis was that there is no difference in clinical and radiographic success rates of indirect pulp treatment performed with Biodentine compared with resin-modified glass ionomer cement (RMGIC) in mature permanent teeth at 12 months of follow-up.

## 2. Materials and Methods

### 2.1. Study Design

We conducted a prospective, split-mouth observational study in adults with deep carious lesions in mature permanent teeth to compare IPT outcomes using Biodentine (Septodont, Saint-Maur-des-Fosses, France) and a resin-modified glass ionomer cement (Fuji II LC, GC Corporation, Tokyo, Japan). The manuscript was prepared in accordance with the STROBE (Strengthening the Reporting of Observational Studies in Epidemiology) guidance for observational studies.

### 2.2. Setting, Ethical Approval, and Study Period

The study started in September 2023 and is ongoing, being conducted at the Denta Aur Dental Medicine Clinic (Târgu Mureș, Romania). The planned follow-up period is 24 months; the present report summarizes the interim results for 12 months. Ethical approval was granted by the Denta Aur Clinic Ethics Committee (No. 031/14 August 2023). The study was conducted in line with the Declaration of Helsinki, and participants provided written consent prior to enrolment.

### 2.3. Participants (Split-Mouth Design)

Recruitment. Patients were consecutively screened among adults attending the clinic for routine dental care. Eligible patients were informed about the study, and those who agreed to participate provided informed written consent before enrollment. Each participant contributed one pair of teeth (two lesions) to limit within-patient clustering.

Inclusion criteria—patient level: Age 25–60 years; willingness to attend follow-up visits at 1, 6, and 12 months; possibility of rubber dam isolation; stable general health; written informed consent. This age range was selected to focus on adult patients with mature permanent teeth while limiting the potential influence of age-related pulpal changes. Younger individuals may exhibit greater pulpal regenerative capacity, whereas advanced pulpal fibrosis and calcification become more common in older patients and may influence vitality responses and treatment outcomes.

Inclusion criteria—tooth/lesion level (pair): Two posterior permanent teeth (molars or premolars) in the same patient, each with a deep carious lesion (radiographically in the inner third of dentin or ≤1 mm from the pulp chamber); vital pulp (at most reversible pulpitis, no spontaneous pain); no apical signs; comparable pair (ideally contralateral homologous teeth, same cavity type, and similar Remaining Dentin Thickness—RDT; if not possible, RDT difference ≤ 0.2–0.3 mm on standardized bitewing/periapical radiographs).

Exclusion criteria (patient and/or tooth): Irreversible pulpitis/necrosis; fistula/swelling/pathologic mobility; extensive restorations or fractures; inability to achieve rubber dam isolation; uncontrolled systemic conditions; allergies; previous endodontic treatment in eligible teeth.

Protocol deviation—Accidental pulp exposure. Because IPT avoids intentional pulp exposure, any accidental exposure in either tooth led to management outside the study (standard vital pulp therapy), and the pair was excluded from complete-pair analyses.

### 2.4. Allocation of Materials and Examiner Blinding

In each participant, one tooth received Biodentine and the contralateral (or comparable) tooth received RMGIC, according to the split-mouth design. Material assignment was predetermined within each pair to ensure both materials were represented in the same participant; no random sequence was used. Operators could not be blinded; however, clinical and radiographic evaluations were performed by two independent, blinded examiners. Any discrepancies were resolved by consensus with a third evaluator.

### 2.5. Interventions (Symmetrical in Both Teeth)

1. Local anesthesia, rubber dam isolation, field disinfection.
2. Selective caries removal: Lateral walls to hard dentin; pulpal wall to firm (leathery) dentin, avoiding exposure.
3. The cavity was disinfected with 2% chlorhexidine for 20 s and gently dried.
4. Application of a 1–1.5 mm liner according to allocation: Test—Biodentine^®^ (Septodont, Saint-Maur-des-Fosses, France); Control—RMGIC (Fuji II LC, GC Corporation, Tokyo, Japan). Biodentine was prepared according to the manufacturer’s instructions by mixing the capsule in an amalgamator and placed directly over the remaining dentin layer in a thickness of approximately 1–1.5 mm. The material was allowed to set before placement of the definitive restoration. For the control group, resin-modified glass ionomer cement was applied as a liner of similar thickness and light-cured according to the manufacturer’s recommendations.
5. The definitive composite restoration was placed immediately using the same closed-sandwich protocol in both teeth to standardize coronal sealing. Adhesive procedures were performed following standard etch-and-rinse protocol, and the composite resin was placed incrementally and light-cured to ensure adequate polymerization and marginal adaptation.
6. Postoperative instructions: Analgesics as required.

### 2.6. Assessments, Standardization, and Calibration

Criteria for success/failure.

Clinical success was defined as the absence of spontaneous pain, a normal response to vitality testing (cold/EPT) without lingering symptoms, negative percussion/palpation, and a functional tooth with an intact restoration. Radiographic success was defined as the absence of new or progressive periapical radiolucency, periodontal ligament widening, or signs of external or internal root resorption. Failure was recorded when persistent pain suggestive of pulpal disease, loss of vitality/necrosis, positive percussion or palpation findings, radiographic signs of periapical pathology or loss of restoration integrity was present. When such findings occurred, appropriate further treatment, including endodontic therapy when clinically indicated, was provided. Accordingly, the primary outcome of the study was defined as composite clinical and radiographic success at 12 months, representing the absence of pulpal or periapical pathosis, as determined by maintenance of pulp vitality, absence of clinical symptoms, intact restoration, and absence of radiographic signs of periapical pathology.

Standardization of investigations.

Periapical radiographs were obtained using the paralleling technique with a dedicated holder and standardized exposure parameters; radiation protection complied with the ALARA principle (As Low As Reasonably Achievable), with image acquisition limited to clinically necessary time points. Radiographs were mandatory at baseline and 12 months and recommended at 6 months. Restoration quality was evaluated using FDI criteria and USPHS (United States Public Health Service) criteria (ordinal scores). Discoloration was assessed by spectrophotometer (ΔE) when available or by standardized visual scoring (Table 1).

Training and calibration of examiners.

Two evaluators were trained on a pilot set and calibrated before the study. For binary variables (presence/absence of apical lesion), Cohen’s κ was used; for ordinal variables (FDI/USPHS scores), weighted κ (quadratic weighting) was used; and for continuous variables (ΔE), the intraclass correlation coefficient—ICC (2,1) (two-way random, absolute agreement)—was calculated. Predefined reliability targets were inter-κ ≥ 0.70 (preferably ≥0.80), intra-κ ≥ 0.80, and ICC ≥ 0.75. Images/photographs were coded and presented in randomized order; evaluators were blinded to the material and patient identity.

Calibration results.

- Binary variables (apical lesion): Inter-examiner κ = 0.82, intra-examiner κ = 0.88.
- Ordinal variables (FDI/USPHS): Weighted inter-κ = 0.79; intra-κ = 0.86.
- Continuous variables (ΔE): ICC (2.1) = 0.80.

All reliability metrics met the predefined targets.

## 3. Results

**Figure 1:**
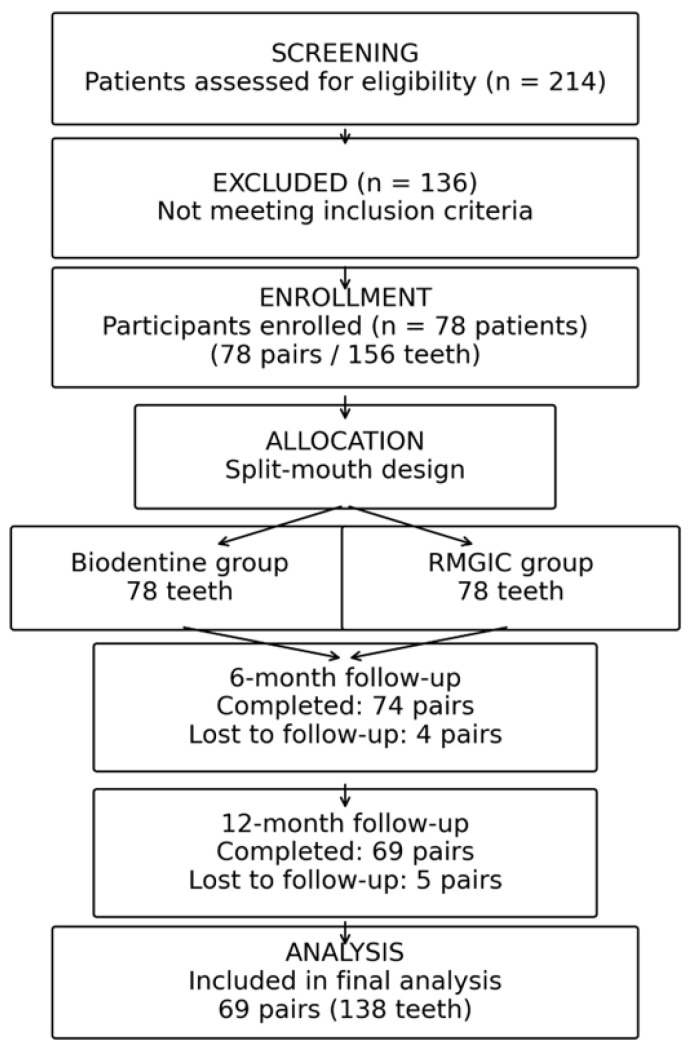
Participant inclusion and follow-up.

Of the 214 patients assessed during the study period, 78 met the inclusion criteria and were enrolled, contributing 78 pairs (156 teeth). Follow-up assessments were completed for 74 pairs at 6 months (94.9%) and for 69 pairs at 12 months (88.5%). Attrition (*n* = 9; 11.5%) was primarily related to missed scheduled visits (*n* = 7) and patient relocation (*n* = 2). All enrolled participants were included in the analyses, with additional evaluations performed on cases with complete follow-up at each assessment point. Participant flow and follow-up are presented in Figure 1.

**Baseline characteristics**. The mean participant age was 38.2 ± 9.1 years, with males accounting for 52.6% of the cohort. Most lesions were occlusal (60.3%), and the median remaining dentin thickness (RDT) at baseline was 0.70 mm (IQR 0.50–0.90). Baseline demographic and lesion-related variables were comparable between the two material groups (Table 2).

Examiner calibration demonstrated satisfactory reliability across all outcome measures. For binary radiographic endpoints, inter- and intra-examiner agreement reached κ = 0.82 and κ = 0.88, respectively. Ordinal FDI/USPHS scores showed weighted κ values of 0.79 (inter-examiner) and 0.86 (intra-examiner), while color change measurements (ΔE) achieved an ICC (2,1) of 0.80. These values exceeded the predefined reliability thresholds, supporting the consistency of outcome assessment.

**Overall clinical and radiographic outcomes**. At both follow-up intervals, combined clinical and radiographic success—defined by absence of spontaneous pain, normal pulp vitality response, negative percussion/palpation, intact restoration, and lack of periapical pathology—was frequently observed in both groups.

At 6 months, success was recorded in 70/74 Biodentine^®^ teeth (94.6%; 95% CI 86.7–98.5) and 69/74 RMGIC teeth (93.2%; 95% CI 84.9–97.8). At 12 months, corresponding values were 65/69 (94.2%; 95% CI 85.6–98.4) for Biodentine^®^ and 63/69 (91.3%; 95% CI 81.0–96.5) for RMGIC. Differences between materials were small, confidence intervals overlapped, and statistical testing did not indicate significant group differences (*p* = 0.75 at 6 months; *p* = 0.73 at 12 months) (Table 3).

### Secondary Outcomes

New apical radiolucency or periodontal ligament widening occurred infrequently and with similar distribution between groups (≤6% at both follow-ups). Postoperative pain assessed at 24 h was lower in the Biodentine^®^ group at both 6 and 12 months (median VAS 2 vs. 3; Wilcoxon *p* = 0.04 and *p* = 0.03), while pain scores converged to ≤1 by day 7 in both groups. Restoration integrity, assessed using FDI/USPHS criteria, was maintained in more than 90% of cases at both timepoints, without material-related differences. Tooth discoloration remained minimal and clinically negligible, with mean ΔE values below 1.5 at 6 and 12 months. No severe adverse events were recorded; transient cold sensitivity during the first postoperative week was reported in fewer than 15% of cases in both groups.

Exploratory analyses. Exploratory subgroup analyses did not reveal differences between age categories (25–40 vs. 41–60 years) or lesion locations (occlusal vs. proximal). No significant material-by-subgroup interactions were identified using generalized estimating equation (GEE) models.

**Figure 2:**
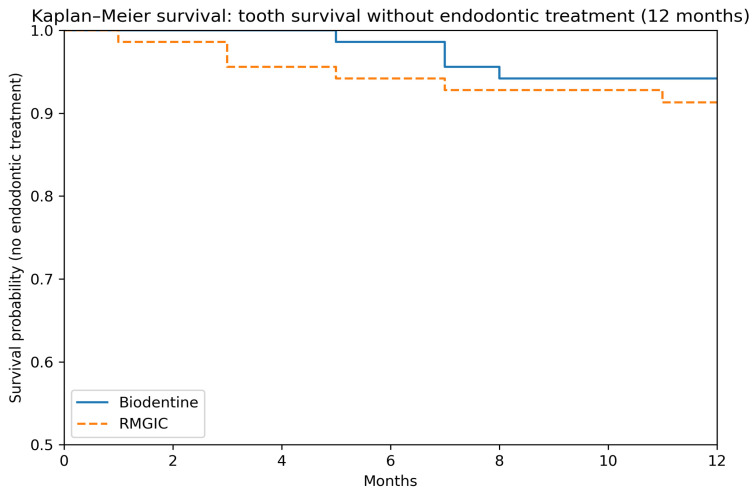
Kaplan–Meier curves illustrating maintenance of tooth vitality without endodontic treatment during 12 months of follow-up. Tick marks indicate censored observations. Curves remained largely overlapping, showing no significant difference between groups.

Kaplan–Meier analysis showed comparable maintenance of tooth vitality without endodontic intervention in both treatment groups over the 12-month period. Survival curves largely overlapped, and the log-rank test did not demonstrate a significant difference between materials (*p* = 0.50) (Figure 2).

## 4. Discussion

### 4.1. Minimally Invasive Treatment Paradigm

The findings of the present study support the minimally invasive management of deep carious lesions and are consistent with the growing body of evidence favoring indirect pulp treatment. Maintaining pulp vitality is a central objective of contemporary dentistry and aligns with the principles of minimally invasive treatment. Current clinical guidelines emphasize selective caries removal rather than complete excavation in deep lesions, primarily to reduce the risk of pulp exposure and preserve sound tooth structure [13,14]. In this context, indirect pulp treatment (IPT) has been increasingly supported as a predictable therapeutic option, with systematic reviews and meta-analyses reporting favorable outcomes for pulp vitality preservation when appropriate case selection and operative protocols are applied [15,16].

Beyond the biological rationale, IPT is also consistent with preventive and cost-containment frameworks in oral healthcare. Given the substantial global economic burden of dental diseases, conservative strategies that prevent escalation toward endodontic and prosthetic treatments are of relevance [6,17]. Together, these considerations position IPT as a clinically and economically sound approach within modern restorative dentistry.

### 4.2. Clinical and Radiographic Success of IPT with Biodentine and RMGIC

In the present study, both Biodentine and resin-modified glass ionomer cement (RMGIC) demonstrated high clinical and radiographic success rates at the 12-month follow-up, with no clinically meaningful differences between the two groups. These findings are consistent with previously published clinical data indicating that IPT can achieve success rates exceeding 85–90% when fundamental operative principles are respected [15,18,19].

Although Biodentine showed a slight, non-significant tendency toward improved outcomes, this observation should be interpreted cautiously and does not indicate material superiority. Rather, it supports the concept that, in mature permanent teeth, the success of IPT is primarily determined by controlled caries excavation and an effective coronal seal, with the liner material representing one component of a broader biological and restorative protocol.

### 4.3. Biodentine Versus Other Bioactive Materials

Traditional liner materials such as calcium hydroxide have historically been used in vital pulp therapy due to their antibacterial properties and ability to induce reparative dentin formation. However, their high solubility and lack of adhesion to dentin have been associated with microleakage and compromised long-term outcomes [5,20]. Resin-modified glass ionomer cements were developed to address some of these limitations by providing chemical adhesion to dentin and fluoride release, although they remain largely passive materials from a biological standpoint [3,21].

Calcium silicate–based materials, including Biodentine, were introduced as bioactive alternatives capable of releasing calcium and hydroxyl ions and promoting the formation of a mineralized interface at the dentin–pulp complex [8,19]. Histological and experimental studies have demonstrated more favorable pulpal responses and higher-quality reparative dentin formation when compared with traditional liners [22,23,24]. Recent clinical evidence further suggests that calcium silicate–based materials may offer advantages over calcium hydroxide in vital pulp therapy, although these benefits appear to be material- and context-dependent [25].

### 4.4. Postoperative Pain and Material Biocompatibility

Postoperative pain represents an important short-term clinical outcome, as it reflects both pulpal response and material biocompatibility. In our study, teeth treated with Biodentine exhibited lower pain scores at 24 h compared with those lined with RMGIC, although this difference was transient and did not persist beyond the first postoperative week.

Previous studies have reported that calcium silicate–based materials are associated with a milder inflammatory response and more favorable early pulpal healing, which may explain the reduced immediate postoperative discomfort observed in the Biodentine group [14,22,26]. The release of calcium ions and the alkaline environment created at the dentin–pulp interface is thought to modulate inflammation and support early tissue repair. Nevertheless, the convergence of pain scores by day 7 indicates that both materials ultimately provide an adequate biological environment for pulp recovery when used in IPT. From a clinical perspective, the observed reduction in early postoperative pain should be interpreted as a short-term comfort benefit rather than a determinant of long-term treatment success. Similar findings have been reported in previous clinical investigations, where early postoperative symptoms differed between materials without influencing pulp vitality or survival at later follow-up intervals [27].

### 4.5. Radiographic Outcomes, Restoration Integrity, and Tooth Survival

Radiographic evaluation remains a cornerstone for assessing the success of indirect pulp treatment. In the present study, the incidence of new periapical radiolucencies or periodontal ligament changes was low and comparable between groups throughout the 12-month follow-up period, in agreement with previous reports on IPT outcomes in permanent teeth [28]. Kaplan–Meier survival analysis further demonstrated comparable maintenance of pulp vitality and tooth survival in both groups, with cumulative survival rates exceeding 90%. This observation reinforces the concept that IPT offers a predictable alternative to more invasive endodontic procedures when applied in teeth with deep carious lesions [18,29]. Importantly, restoration integrity plays a decisive role in long-term treatment success. Evidence consistently shows that inadequate coronal sealing increases the risk of bacterial microleakage and pulpal breakdown, regardless of the liner material used [30,31]. In the present study, standardized restorative protocols were employed, which likely contributed to the favorable radiographic and survival outcomes observed.

Discoloration associated with liner materials represents an additional clinical consideration. Previous investigations have shown that Biodentine is less prone to inducing coronal discoloration compared with other calcium silicate–based materials, such as mineral trioxide aggregate, supporting its use in esthetically relevant areas [8,23,32].

### 4.6. Prognostic Factors and Clinical Relevance

Several prognostic factors may influence the outcome of vital pulp therapy in permanent teeth. Age-related changes in pulpal tissue, including reduced cellularity, increased fibrosis, and diminished reparative capacity, may limit the biological advantages of bioactive materials in mature teeth compared with younger populations [7,33]. This consideration may partly explain why Biodentine did not demonstrate clear superiority over RMGIC in the adult cohort investigated. From a pragmatic standpoint, both Biodentine and RMGIC can be considered defensible liner materials for IPT in mature permanent teeth. While Biodentine offers specific biological advantages, RMGIC provides predictable handling, chemical adhesion, and fluoride release, supporting its continued use in routine clinical practice [3]. These findings suggest that clinical decision-making should prioritize diagnosis, case selection, and operative technique rather than reliance on a specific material. Beyond individual clinical outcomes, the use of IPT has broader economic and public health implications. Conservative management of deep carious lesions has been shown to reduce long-term treatment costs and the need for complex endodontic or prosthetic interventions, supporting its integration into cost-effective oral healthcare strategies [34,35].

### 4.7. Strengths and Limitations

The strengths of this study include its split-mouth design, which minimized inter-individual variability, and the use of blinded clinical and radiographic outcome assessment. In addition, the application of standardized operative and restorative protocols enhanced the internal validity of the findings.

Several limitations should be acknowledged. The sample size was moderate, and the present analysis represents interim results from an ongoing 24-month observational study. Consequently, longer follow-up is required to confirm the durability of these outcomes. Furthermore, the single-center design may limit generalizability, and multicenter studies with extended follow-up periods are warranted to further validate these results.

The present findings may serve as preliminary data for sample size estimation in future randomized clinical trials designed to further compare Biodentine and RMGIC in indirect pulp treatment.

## 5. Conclusions

In mature permanent teeth with deep carious lesions, indirect pulp treatment resulted in favorable clinical and radiographic outcomes at 12 months when performed with either Biodentine or resin-modified glass ionomer cement. The absence of meaningful differences in overall success between materials indicates that pulp vitality preservation can be achieved through different liner strategies when minimally invasive principles and effective coronal sealing are respected. Biodentine was associated with reduced early postoperative discomfort; however, this short-term benefit did not translate into superior mid-term outcomes. From a clinical perspective, these findings suggest that material selection alone is unlikely to determine treatment success, which appears to depend primarily on case selection, operative protocol, and restoration quality.

As interim results from an ongoing observational study, the present data should be interpreted with caution. The planned 24-month follow-up will be essential to confirm the durability of these outcomes and to further inform clinical decision-making in adult vital pulp therapy.

## Figures and Tables

**Table 1:** Schedule of assessments in the split-mouth study.

Time Point	Clinical Exam	Pulp Vitality (Cold/EPT)	Pain (VAS)	Perc./Palp.	Periapical RX	Restoration (FDI/USPHS)	Other Obs.
Baseline	✔	✔	–	✔	✔	✔	Pair assignment (split-mouth)
1 month	✔	✔	✔ (24 h, 7 d)	✔	If symptoms	✔	Discoloration
6 months	✔	✔	✔	✔	✔ (rec.)	✔	Discoloration
12 months	✔	✔	✔	✔	✔ (mand.)	✔	Primary outcome

Note: ✔ = assessment performed; VAS = Visual Analog Scale; FDI/USPHS = criteria for restoration evaluation.

**Table 2:** Baseline characteristics of the study participants.

Characteristic	Value (*n* = 78)	Notes
Age, years (mean ± SD)	38.2 ± 9.1	Range 25–60
Male, *n* (%)	41 (52.6)	—
Female, *n* (%)	37 (47.4)	—
Occlusal lesions, *n* (%)	94/156 (60.3)	Comparable pairs
Proximal lesions, *n* (%)	62/156 (39.7)	—
Molars, *n* (%)	102/156 (65.4)	—
Premolars, *n* (%)	54/156 (34.6)	—
Maxilla, *n* (%)	85/156 (54.5)	—
Mandible, *n* (%)	71/156 (45.5)	—
RDT, mm (median, IQR)	0.70 (0.50–0.90)	Measured at baseline

**Table 3:** Clinical and radiographic outcomes at 6 and 12 months.

Outcome	Biodentine (6 Months)	RMGIC (6 Months)	*p*-Value	Biodentine (12 Months)	RMGIC (12 Months)	*p*-Value
Clinical + radiographic success	70/74 (94.6%)	69/74 (93.2%)	0.75	65/69 (94.2%)	63/69 (91.3%)	0.73
New apical radiolucency/PDL widening	1 (1.4%)	2 (2.7%)	0.56	2 (2.9%)	4 (5.8%)	0.68
VAS pain 24 h (median, IQR)	2 (1–3)	3 (2–4)	0.04	2 (1–4)	3 (2–5)	0.03
VAS pain 7 days (median, IQR)	≤1	≤1	NS	≤1	≤1	NS
Restoration integrity (FDI A/B)	>90%	>90%	0.82	>90%	>90%	0.74
Discoloration ΔE (mean ± SD)	1.1 ± 0.5	1.3 ± 0.6	0.27	1.2 ± 0.6	1.4 ± 0.7	0.29
Severe adverse events	0	0	—	0	0	—

## Data Availability

The datasets presented in this article are not readily available because they contain de-identified patient data that are subject to ethical and confidentiality restrictions related to informed consent. Requests to access the datasets should be directed to the corresponding author.
